# Correlation of finger-to-floor distance with the spinal mobility, spinal function indices and initial determination of its optimal cutoff value: a multicentre case–control study

**DOI:** 10.3389/fmed.2023.1135748

**Published:** 2023-06-22

**Authors:** Genggeng Guo, Yulu Zhang, Diantian Lin, Zhihan Chen, Qing Yan, Fei Gao, Yanfang Wu, Juanjuan He, Da Chen, Zugang Xie, Feng Huang, Shengli Zhang

**Affiliations:** ^1^Department of Rheumatology, Shengli Clinical Medical College of Fujian Medical University, Fuzhou, Fujian, China; ^2^Department of Rheumatology, People's Hospital of Longyan, Longyan, Fujian, China; ^3^2018 Grade of Department of Preventive Medicine, School of Public Health, Fujian Medical University, Fuzhou, Fujian, China; ^4^Department of Rheumatology and Immunology, The First Medical Center, Chinese PLA General Hospital, Beijing, China

**Keywords:** ankylosing spondylitis, finger-to-floor distance, spinal mobility, receiver operating characteristic curves, cutoff value

## Abstract

**Objective:**

To identify the correlation between finger-to-floor distance(FFD) and the spinal function indices and disease activity scores of ankylosing spondylitis (AS) via a multicentre case–control study, and to calculate the optimal cutoff value of FFD using statistical methods.

**Methods:**

Patients with AS and healthy individuals were recruited, and the FFD and other spinal mobility values were measured. The correlation between the FFD and the Bath Ankylosing Spondylitis Metric Index (BASMI), Bath Ankylosing Spondylitis Disease Activity Index (BASDAI), Bath Ankylosing Spondylitis Functional Index (BASFI) was analyzed using Spearman rank correlation analysis. Receiver operating characteristic (ROC) curves of FFD stratified by gender and age were drawn and their optimal cutoff values were determined.

**Results:**

A total of 246 patients with AS and 246 healthy subjects were recruited. The FFD was strongly correlated with BASMI (*r* = 0.72, *p* < 0.001), moderately correlated with BASFI (*r* = 0.50, *p* < 0.001) and weakly correlated with BASDAI (*r* = 0.36, *p <* 0.001). The lowest cutoff value of the FFD was 2.6 cm while the highest was 18.4 cm. Moreover, the FFD was significantly correlated with sex and age.

**Conclusion:**

There exists a strong correlation between the FFD and spinal mobility, a moderately correlation and function, which provides reliable data for the evaluation of patients with AS in clinical settings and the rapid screening of low back pain-related diseases in the general population. Furthermore, these findings have clinical potential in improving the missed diagnosis or delayed diagnosis of low back pain.

## Introduction

Ankylosing spondylitis (AS) is a systemic and progressive rheumatic disease characterized by chronic inflammation of the spine and sacroiliac joints. Spinal structural damage is one of the important characteristics of AS. Spinal mobility and spinal function are important indicators for the diagnosis, disease progression, efficacy and prognosis of patients with AS (1–3). Furthermore, early abnormal spinal mobility has been speculated to predict spinal fusion in patients with AS (4). Currently, Bath Ankylosing Spondylitis Measurement Index (BASMI) is the internationally recognized method to assess the spinal mobility and functional status in patients with AS (5). However, BASMI measures various items and is time-consuming, estimating approximately 10–15 min for a trained rheumatologist to evaluate a patient (6). The subtle differences in the scoring method of the various versions in BASMI make it difficult to be adopted by other specialists, such as orthopedics, pain, general practice or community health census volunteers. Additionally, generalizing the BASMI scores over various medical specialities or workers for the early screening of AS or other aetiologies of low back pain is unsuitable and difficult (7–9). Therefore, developing a fast, simple and reliable method for spinal mobility measurement that can not only accurately assess the spinal function status of patients with AS in rheumatology specialities but also effectively be applied to other clinical specialities or community preliminary screening of AS or other low back pain diseases is vitally important.

The finger-to-floor distance (FFD) and the modified Schober’s test are both commonly applied clinical assessments for spinal mobility, evaluating the involvement of various regions, including the spine, hip joints, shoulder joints, and knee joints (10). The modified Schober’s test assesses spinal mobility by measuring the increased distance between two marked points on the patient’s lower back before and after bending, and due to its high precision, it is included in the ASAS core set and BASMI assessment items. However, the test involves multiple steps, is time-consuming, and current studies indicate that the primary reason for normal BASMI scores exceeding 0 is abnormality in the modified Schober’s test, with its reference values being a subject of considerable controversy. The FFD, though not included in the ASAS core set and BASMI assessment items, has the advantages of being simple, rapid, and easily mastered. It can ascertain improvements in spinal mobility in AS patients during baseline or post-treatment periods in just a few seconds, as well as being used for preliminary screening for other causes of low back pain to prevent diagnostic delay. Previous studies have indicated that FFD is a reliable and sensitive measurement for assessing changes in spinal mobility in AS patients (11–16).

Currently, a unified reference value range for FFD measurement does not exist. The values for FFD are 0 or close to 0, which often indicate a good spinal mobility, whereas higher values mostly indicate an abnormal pathological spinal mobility. The lack of research on the normal value and the optimal cutoff value of the FFD hinders the interpretation and significance of FFD measurement analyses. This study aims to measure and analyze the correlation between FFD and BASMI, BASDAI, BASFI, CRP, ESR and other indicators. Additionally, it aims to determine the optimal cutoff value of FFD stratified by gender and age ROC curves.

## Materials and methods

### Design and objects of the study

This study was a multicentre case–control study. Patients with AS were recruited from the Rheumatology Outpatient Department and wards of Fujian Provincial Hospital and People’s Hospital of Longyan City, Fujian Province. The FFD and spinal mobility were assessed, the demographic characteristics, BASDAI and BASFI scores were evaluated and the related hematological indicators, including ESR, CRP and HLA-B27, were detected in patients with AS. The inclusion criteria for the patient group were as follows: All patients met the New York Classification Criteria for AS revised in 1984 (17), and the disease duration was ≤10 years. Current treatments for these AS patients include nonsteroidal anti-inflammatory drugs, sulfasalazine, methotrexate, tumor necrosis factor antagonists, interleukin-17A antagonists, etc.The exclusion criteria were as follows: (1) severe joint or spinal pain at the time of the study; (2) previous total hip arthroplasty or severe limitation of hip mobility; (3) history of vertebral fracture, spinal surgery, severe scoliosis, spinal deformity, or complete segmental fusion of the spine; 4) pregnancy or inability to ambulate without assistance or mobility assistive devices.

Healthy control subjects were recruited from Fujian Normal University, Fujian Shipping and Transportation Vocational College and the residential communities. FFD, demographic characteristics and any current or past medical history were evaluated and obtained from the healthy subjects. The inclusion criteria of the control group were as follows: the age and sex ratio were consistent with those of the patient group. The exclusion criteria were as follows: (1) history of back surgery; (2) history of low back pain in the past 3 months; (3) history of inflammatory rheumatic disease; (4) pregnancy of more than 3 months; (5) severe scoliosis; (6) symptomatic disc disease at the time of evaluation; (7) hip replacement or any known hip disease (8) acute or chronic respiratory tract infection, acute or chronic chest or back pain. In accordance with the Declaration of Helsinki, the study was approved by the Ethics Committee of Fujian Provincial Hospital (Approval ID: K-2018-10-014), and all participants provided written informed consent.

### Grouping and sample size determination

A previous study reported that the age group with a high incidence of AS was 25–34 years (18). Therefore, we divided the male and female subjects into three groups: <25 years, 25–34 years (high incidence age group) and >34 years. The best critical value of FFD for different gender and age groups was calculated. We set a minimum acceptable area of 0.7 under the ROC curve, a Type I error of *α* = 0.05, a Type II error of *β* = 0.10 and a healthy to patient group ratio of 1. It was estimated that at least 82 individuals would need to be included in each group, with a total of 492 individuals in the study.

### Measurements of spinal mobility and data collection

Measurements of spinal mobility included FFD, tragus-to-wall distance, lumbar lateral flexion, modified Schober test, cervical rotation in the sitting position and intermalleolar distance in the lying position. All items were measured twice and averaged. Cervical rotation was measured in degrees whereas the remaining items were measured in centimetres. The BASMI-10 score was calculated as the average of the five measured item scores. All items were measured according to the methods recommended by the 2009–2010 ASAS Manual, no warm-up activities were conducted before the measurement, and low platform measurements were not used to measure the FFD (19). Two groups of surveyors, each with two surveyors, measured spinal mobility. One group was responsible for measurement while the other was responsible for guiding participants to cooperate in the measurement and verifying the accuracy of measurement technology. Before the start of the survey, the four surveyors were trained for 2 days, and the survey was initiated after all the surveyors were qualified. The first 25 participants were measured simultaneously by the two groups of surveyors, and the inter-surveyors’ errors were assessed by calculating the intraclass correlation coefficient (ICC). After 24 h, each measure of spinal mobility in these 25 participants was re-measured at the same time as the previous day, and the intra-surveyors’ errors were assessed using ICC.

The demographic characteristics, BASDAI and BASFI scores of all patients with AS were investigated, and the ESR, CRP and HLA-B27 of patients with AS were detected using blood sampling.

### Statistical analysis

The SPSS 26.0 software was used for data statistical processing. Arithmetic mean ( $\bar{X}$ ) and standard deviation (*SD*) were calculated for normal distribution data, whereas median (M) and interquartile interval (IQR) were calculated for non-normal distribution data. The FFD of the patient group and the healthy control group were stratified by age and sex. Spearman’s rank correlation coefficient was used to analyze the correlation between the FFD and BASMI, BASDAI, BASFI, CRP and ESR. The correlations were classified by *r* value as: no correlation (*r* < 0.20), general correlation (0.20 ≤ *r* < 0.40), moderate correlation (0.40 ≤ *r* < 0.70) and strong correlation (*r* ≥ 0.70) (20).

Logistic regression analysis was employed to examine the relationship between demographic variables and the FFD measurements. Variables with statistically significant differences in univariate analysis were used as independent variables for subsequent multivariate logistic regression analysis. A value of *p* less than 0.05 was considered statistically significant. Additionally, the area under the curve (AUC), standard error (SE) and Youden’s index (Youden’s index = sensitivity + specificity-1) were calculated. The optimal cutoff value of the FFD was defined as the maximum Youden index and considered statistically significant at *p* < 0.05.

### Patient and public involvement

Patients and/or the public were not involved in designing, conducting, reporting, or disseminating the plans of this research.

## Results

A total of 246 patients with AS and 246 healthy individuals were enrolled between January 2021 and January 2022, with equal numbers of men and women in each group. The patient group had an average age of 29.0 years (23.0, 37.0) and a disease course of 5.0 years (2.5, 7.0). The healthy group had an average age of 30.0 years (20.0, 39.0). The general data of the two groups were balanced and comparable, with no significant difference in gender and age distribution (Table 1).

**Table 1:** Demographic characteristics of the healthy control group and the patient group.

Characteristics	Healthy group (*n* = 246)	Patient group (*n* = 246)	*Z*/*X*^2^	Value of *p*
Age (years)^a^	30.0 (20.0, 39.0)	29.0 (23.0, 37.0)	−0.304	0.761
Height (cm)^a^	170.0 (165.0, 174.0)	170.0 (164.0, 175.0)	−0.861	0.389
Weight (kg)^a^	62.0 (55.0, 70.0)	63.0 (55.0, 72.0)	−1.648	0.099
FFD (cm)^a^	5.9 (0, 11.5)	14.3 (7.4, 21.0)	–	–
BASMI^a^	–	2.0 (1.6, 3.4)	–	–
BASDAI^a^	–	3.7 (2.3, 5.4)	–	–
BASFI^a^	–	1.5 (0.4, 3.8)	–	–
ESR (mm/h)^a^	–	6.0 (3.0, 17.0)	–	–
CRP (mg/L)^a^	–	3.3 (1.0, 12.9)	–	–
Disease course (years)^a^	–	5.0 (2.5, 7.0)	–	–
Morning stiffness time (min)^a^	–	3.0 (0, 10.0)	–	–
HLA-B27: positive, n (%)^b^	–	217 (88.2%)	–	–

^a^M (IQR), ^b^B: Percentage. *p* < 0.05 is considered statistically significant. FFD, finger-to-floor distance; BASMI, Bath Ankylosing Spondylitis Metrology Index; BASDAI, Bath Ankylosing Spondylitis Disease Activity Index; BASFI, Bath Ankylosing Spondylitis Functional Index; CRP, C-reactive protein; ESR, erythrocyte sedimentation rate.

Table 2 shows the intra-and inter-observer ICC of FFD and BASMI. Intra-and inter-observer ICC was high for all items, and the intra-and inter-observer ICC of FFD better than that of BASMI.

**Table 2:** The inter-observer and intra-observer ICC of FFD and BASMI.

Items of the survey	Inter-observers ICC	Value of *p*	Intra-observers ICC	Value of *p*
FFD	0.961	<0.001	0.948	<0.001
BASMI	0.945	<0.001	0.934	<0.001

ICC, interclass correlation coefficient; FFD, finger-to-floor distance; BASMI, Bath Ankylosing Spondylitis Metrology Index.

Table 3 shows the correlation between FFD and BASMI, BASDAI, BASFI, CRP, ESR and other indicators analyzed using Spearman’s correlation. FFD revealed a strong correlation with BASMI, a medium correlation with BASFI and a general correlation with BASDAI and ESR. BASMI had a moderate correlation with BASFI and a general correlation with BASDAI and ESR. However, no correlation between FFD, BASMI and CRP was observed.

**Table 3:** Analysis of Spearman’s correlation between FFD and BASMI, BASDAI, BASFI, CRP, ESR.

Items of the survey	BASMI	BASDAI	BASFI	ESR	CRP
FFD	*r* = 0.72^**^	*r* = 0.36^**^	*r* = 0.50^**^	*r* = 0.23^**^	*r* = 0.10
BASMI	–	*r* = 0.35^**^	*r* = 0.40^**^	*r* = 0.30^**^	*r* = 0.07

*p < 0.05; **p < 0.01. No correlation (*r* < 0.20), general correlation (0.20 ≤ *r* < 0.40), moderate correlation (0.40 ≤ *r* < 0.70) and strong correlation (*r* ≥ 0.70). FFD, finger-to-floor distance; BASDAI, Bath Ankylosing Spondylitis Disease Activity Index; BASFI, Bath Ankylosing Spondylitis Functional Index; BASMI, Bath Ankylosing Spondylitis Metrology Index; CRP, C-reactive protein; ESR, erythrocyte sedimentation rate. All the *p* values with correlations were less than 0.01.

Table 4 presents the results of the multivariate logistic regression analysis of abnormal FFD measurements in healthy control subjects and AS patients. Univariate regression analysis of the healthy control group found that age, gender, height, and weight were all related to abnormal FFD measurements. Multivariate logistic regression analysis with the presence of abnormal FFD as the dependent variable showed that age and gender were independently associated with abnormal FFD, while height and weight were not. Univariate regression analysis of the patient group found that age, gender, and disease duration were related to abnormal FFD. Multivariate logistic regression analysis with the presence of abnormal FFD as the dependent variable showed that age and gender were independently associated with abnormal FFD, while disease duration was not.

**Table 4:** Results of the multivariable logistic regression analysis for abnormal finger-to-floor distance.

Group		Standard error	Wald	OR (95% CI)	Value of *p*
Healthy group	Age	0.018	42.634	1.128(1.088–1.169)	<0.001
	Gender	0.364	7.577	2.721(1.334–5.549)	0.006
	Height	0.030	0.122	1.010(0.953–1.071)	0.727
	Weight	0.018	0.408	1.012(0.976–1.048)	0.523
Patient group	Age	0.200	10.341	1.067(1.025–1.109)	0.010
	Gender	0.319	9.919	2.734(1.462–5.112)	0.020
	Course of disease	0.058	1.782	1.081(0.964–1.212)	0.182

*p* < 0.05 is considered statistically significant.

**Figure 1:**
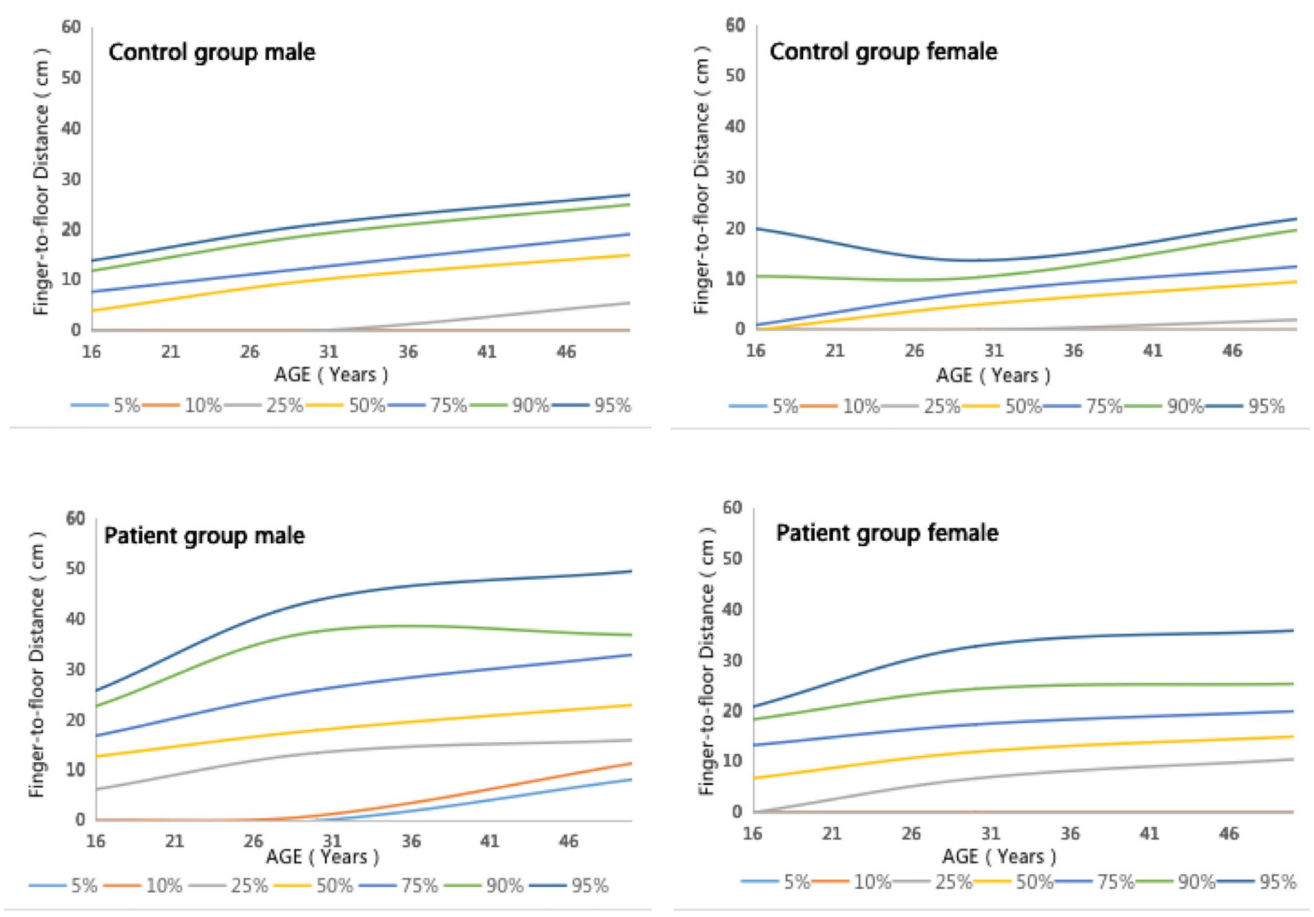
Percentile curves of the FFD measurement values for males and females, stratified by age.

Figure 1 presents the percentile curves of FFD measurements for males and females, stratified by age. In both the healthy control group and the patient group, FFD increases with age, and the FFD for males is greater than that for females.

**Figure 2:**
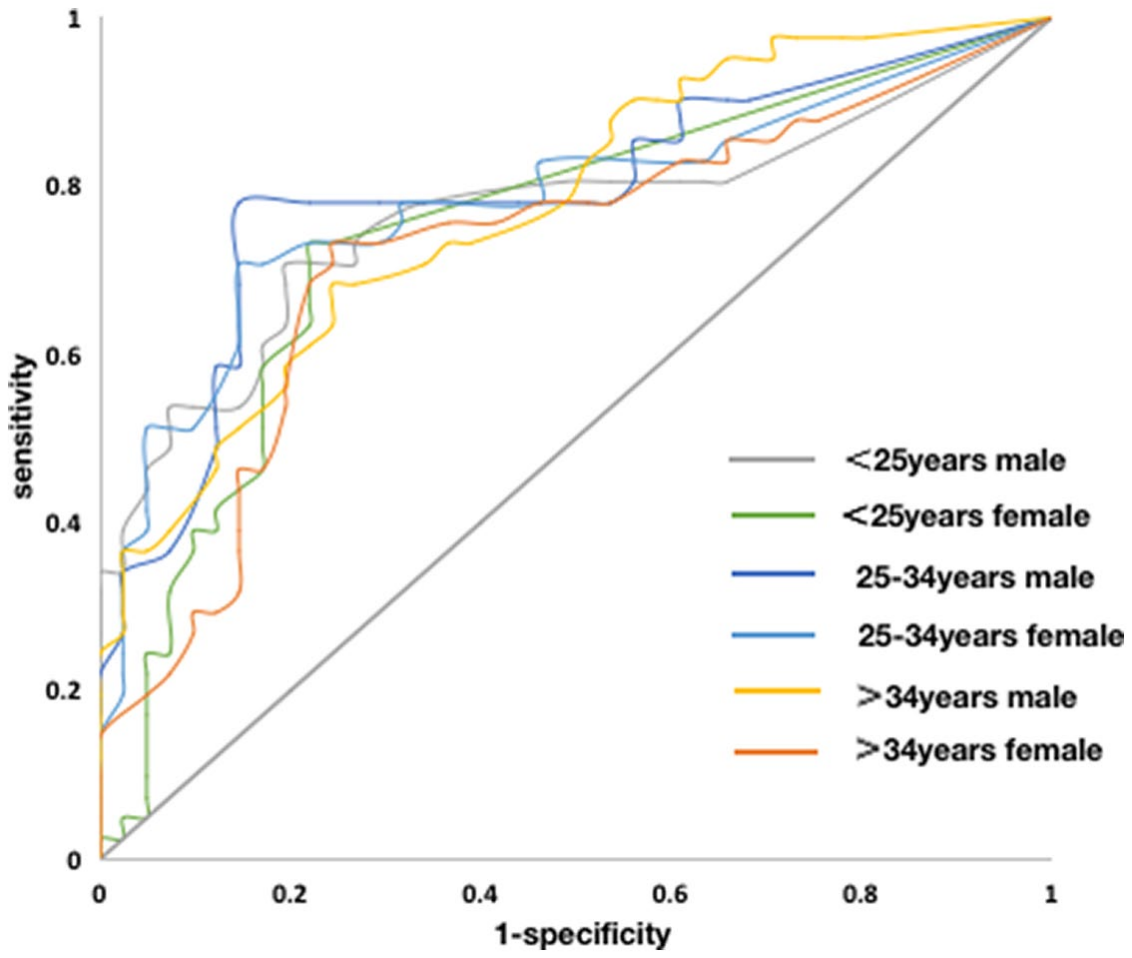
The receiver operating characteristic curve of the FFD stratified by sex and age.

According to the gold standard of the results of clinical diagnosis, the ROC curve of FFD stratified by sex and age is drawn with sensitivity as the ordinate and (1-specificity) as the abscissa. According to the sensitivity and specificity of each critical value in the ROC curve, the Youden index was calculated (Youden index = sensitivity + specificity-1), and the critical value with the highest Youden index was regarded as the optimal cutoff value. The ROC curve of the FFD stratified by sex and age and the optimal cutoff value are presented in Figure 2 and Table 5.

**Table 5:** The optimal cutoff value of the FFD.

	Area under the curve	95% confidence interval	Sensitivity	Specificity	Youden index	Optimum cutoff value (cm)
<25 years male	0.767	0.660–0.874	70.7%	80.5%	0.512	8.8
<25 years, female	0.749	0.640–0.858	73.2%	78.0%	0.512	2.6
25–34 years, male	0.791	0.690–0.893	78.0%	85.4%	0.634	13.3
25–34 years, female	0.783	0.679–0.886	70.7%	85.4%	0.561	9.3
> 34 years, male	0.776	0.677–0.875	68.3%	75.6%	0.439	18.4
>34 years, female	0.724	0.611–0.837	73.2%	75.6%	0.488	12.3

## Discussion

FFD, as a simple, efficient, and easy-to-master method for measuring spinal mobility, is primarily used to evaluate impairment in multiple areas including the spine, pelvis, and knee joints. Numerous studies have recommended the use of FFD in clinical practice to assess spinal mobility in patients with AS (6, 12). In this study, we measured the FFD and analyzed its reliability and correlation with BASMI, BASDAI, BASFI, CRP, ESR and other indicators. On evaluating 246 patients with AS and 246 normal individuals, we preliminarily determined the optimal cutoff value of FFD using a ROC curve. To the best of our knowledge, this is the first comprehensive and detailed study of FFD using a multicentre case–control study design.

By measuring the precise value of FFD and determining its correlation with traditional metrology, such as AS functional indexes and disease activity scores, our study revealed that FFD had a strong correlation with BASMI (*r* = 0.72), a moderate correlation with BASFI (*r* = 0.5). Moreover, it was found that there was and a moderate correlation between BASMI and BASFI (*r* = 0.4). Almodóvar et al. (6) conducted a cross-sectional study of 842 patients with AS and reported a moderate correlation between FFD and BASFI, which was consistent with our findings. In the current study, a strong correlation was observed between FFD and BASMI. Moreover, the correlation between FFD and BASFI (*r* = 0. 5) was better than that of BASMI and BASFI (*r* = 0.4), indicating that FFD had the same high accuracy as BASMI in evaluating the spinal mobility. Measurements of FFD and BASMI were performed by two groups of surveyors. Consistent with the results of Viitanen et al. (11), we also observed that both FFD and BASMI had higher intra- and inter-observer ICCs, and the intra- and inter-observer ICCs of FFD were better than those of BASMI. The ICC values of the FFD measurement were higher than other measurement items of spinal mobility, highlighting that FFD had high reliability and sensitivity. Additionally, FFD and BASMI had higher intra-surveyor and inter-surveyors ICCs, indicating that both FFD and BASMI had better repeatability and consistency between different observers, and the repeatability and consistency between different surveyors of FFD were better than those of BASMI.

On stratifying FFD by age, we observed that FFD increased with age in both the healthy control group and patient group, which is consistent with previous studies (3, 21–23). After stratified comparison by gender, the FFD of men was significantly higher than that of women in both groups. Thus, The incremental impact of age should be considered in the clinical assessment of FFD in older patients with AS, and the differences in physiological characteristics should also be taken into account in the AS assessment of the different genders.

As the FFD was significantly correlated with sex and age, we used the clinical diagnosis as the gold standard to draw the ROC curve of FFD stratified by sex and age and determine the optimal cutoff value. The ROC curve has unique advantages in evaluating the value of clinical diagnostic methods. The more the ROC curve shifted toward the upper left, the larger the area under the curve, which reflects the higher clinical accuracy of the diagnostic method (24). In this study, the area under the FFD curve stratified by gender and age was 0.724–0.791, indicating a high diagnostic accuracy and value. For example, the optimal cutoff value of the FFD for men <25 years was 8.8 cm, indicating that a FFD > 8.8 cm in men <25 years could be correlated with pathologically restricted spinal mobility. Therefore, in clinical settings, it is essential to measure the FFD for individuals with low back pain or other AS-related clinical features. If the FFD is abnormal, careful screening should be performed to prevent the missed diagnosis of AS or other low back pain-related diseases.

This study has various advantages. It investigated both groups, patients with AS and healthy control, simultaneously to ensure a certain degree of sensitivity and specificity. Furthermore, the maximum value of the Youden index as the optimal cutoff value was used to ensure that the determination of the optimal cutoff value was more objective, specific and accurate. The limitations of this study include that, due to the differences in spinal mobility among different ethnic groups, the results of this study may be more applicable to the Asian Mongoloid population. The optimal cutoff values for spinal mobility in other ethnic groups may derive certain referential value from this study. It’s important to emphasize that an abnormal FFD is not a pathological sign specific to AS patients; other diseases causing lower back pain can also result in abnormal FFD. Although the study population includes patients with AS and healthy people, the age range is not comprehensive and the analysis of patients with AS using imaging techniques is insufficient. Therefore, further studies using a larger sample size and age range should be performed to ensure wider generalization.

In conclusion, this study revealed that FFD has a strong correlation with the spinal mobility and function. As a rapid, efficient and reliable method of measurement, FFD can provide reliable data for the assessment of patients with AS in clinical settings and the rapid screening of low back pain-related diseases in the general population. Furthermore, for men and women of all ages whose FFD value reaches or exceeds the optimal cutoff value, it is necessary to determine the presence of pathological spinal diseases to prevent missed diagnosis or delayed diagnosis.

## Data availability statement

The original contributions presented in the study are included in the article/supplementary material, further inquiries can be directed to the corresponding authors.

## Ethics statement

The studies involving human participants were reviewed and approved by the Ethics Committee of Fujian Provincial Hospital (Approval ID: K-2018-10-014) and conducted in accordance with the Declaration of Helsinki (as revised in 2013). The patients/participants provided their written informed consent to participate in this study.

## Author contributions

GG, SZ, and FH: conception and design. ZC: administrative support. DL, YW, and ZX: provision of study materials or patients. YZ, JH, and QY: collection and assembly of data. GG, YZ, and DC: data analysis and interpretation. GG, YZ, DL, ZC, QY, FG, YW, JH, DC, ZX, FH, and SZ: manuscript writing and final approval of manuscript. All authors contributed to the article and approved the submitted version.

## Funding

This study was supported by General program of Fujian Natural Science Foundation (2020J011077) and Longyan Science and Technology Innovation Joint Fund Health Project (2022LYF17002).

## Conflict of interest

The authors declare that the research was conducted in the absence of any commercial or financial relationships that could be construed as a potential conflict of interest.

## Publisher’s note

All claims expressed in this article are solely those of the authors and do not necessarily represent those of their affiliated organizations, or those of the publisher, the editors and the reviewers. Any product that may be evaluated in this article, or claim that may be made by its manufacturer, is not guaranteed or endorsed by the publisher.
